# Society Meetings

**Published:** 1897-07

**Authors:** 


### Society Meetings.

The American Association of Obstetricians and Gynecologists will hold its tenth annual meeting at the Cataract House, Niagara Falls, Tuesday, Wednesday, Thursday and Friday, August 17, 18, 19 and 20, 1897, under the presidency of Dr. James F. W. Ross, of Toronto. The railways have granted reduced fares on the certificate plan to all who attend the meeting; the Cataract House has made a reduction from its regular tariff of charges; the place of meeting is a famous one; the season of the year auspicious, and everything seems to conspire to justify a prediction that this will be a large and interesting meeting of this famous association.

The date of the meeting has been fixed in mid-August, apart from college sessions, during the vacation season, and at a place where many people like to spend a portion of their outing. The climate of Niagara is always desirable during the heated term, the spray from the cataract giving it a healthy moisture and coolness that is at once invigorating and charming. To visit Niagara under the auspices of this association will afford the tourists exceptional opportunities for the enjoyment of a rare and radiant scenery that is the most sublime in the world. One session will be devoted to the exhibition of specimens and giving their histories.

The scientific work of the association will begin on Tuesday morning at 10 o'clock and end Friday at 1 o'clock, and it is expected to so arrange the program as to afford the members opportunity to visit the places of interest each day on the adjournment of the afternoon session. It is expected that the inducements to attend this meeting are such that Fellows will not only come themselves, but bring their families and invite their friends as well to visit the wondrous cataract.

———

THE Buffalo Academy of Medicine held meetings during the month of June as follows :

*Annual Meeting of the Academy.*—Tuesday evening, June 8th. Program : annual address of the president, Dr. Herman Mynter, Prognosis of appendicitis.

*Section on Pathology.*—Tuesday evening, June 15th. Program : Some deformities of the chest in the light of its ancestry and development, by Dr. Woods Hutchinson ; exhibition of gall-stones, by Dr. A. L. Benedict ; exhibition of specimens, by Dr. C. C. Frederick.

*Section on Obstetrics and Gynecology.*—Tuesday evening, June 22d. Program : Puerperal septicemia, by Dr. L. G. Hanley ; Nature of dysmenorrhea, by Dr. M. D. Mann.

———

THE Ohio State Medical Society held its annual meeting at Cleveland, May 19, 20 and 21, 1897. The meeting next year will be held at Columbus on a date to be fixed hereafter. The election of officers resulted as follows : president, Dr. William H. Humiston, of Cleveland ; vice-presidents, T. Clark Miller, M. D., Massillon ; Geo. Mitchell, M. D., Mansfield ; E. H. Hyatt, M. D., Delaware ; D. H. Brinkerhoff, M. D., Fremont ; secretary, John A. Thompson, M. D., Cincinnati ; assistant-secretary, H. M. W. Moore, M. D., Columbus ; treasurer, James A. Duncan, M. D., Toledo ; Editor, R. Harvey Reed, M. D., Columbus.

The Association of Military Surgeons of the United States held a most successful meeting at Columbus, O., May 25, 26 and 27, 1897, under the presidency of Commodore Albert L. Gihon, M. D., Medical Director United States Navy (retired). The election of officers resulted as follows: President, Brigadier-General (retired) Jefferson D. Griffith, Surgeon-General N. G. Mo., Kansas City, Mo.; first vice-president, Major John Van Rensselaer Hoff, Surgeon United States Army, Washington, D. C.; second vice-president, Commander John C. Wise, Medical Inspector United States Navy, Washington, D. C.; secretary, Captain James E. Pilcher, Assistant Surgeon United States Army, Columbus; treasurer, Major James Jay Erwin, Assistant Surgeon-General Ohio National Guard, Cleveland. Kansas City, Mo., was chosen for the next place of meeting.

The American Medical Publishers' Association held its fourth annual meeting at the Hanover, Philadelphia, Monday, May 31, under the presidency of Dr. Landon B. Edwards, of Richmond, editor of the *Virginia Medical Semi-monthly*. The president delivered his address, a number of papers were read and the usual business transacted. It is expected to meet next year at Denver.

